# Intravascular Lithotripsy and Drug-Coated Balloon Angioplasty for Severely Calcified Femoropopliteal Arterial Disease

**DOI:** 10.1177/15266028221075563

**Published:** 2022-02-07

**Authors:** Konstantinos Stavroulakis, Theodosios Bisdas, Giovanni Torsello, Nikolaos Tsilimparis, Sarah Damerau, Angeliki Argyriou

**Affiliations:** 1 Department of Vascular Surgery, St. Franziskus-Hospital GmbH, Muenster, Germany; 2 Department of Vascular Surgery, Ludwig-Maximilians-University Hospital Munich, Munchen, Germany; 3 Department of Vascular and Endovascular surgery, Athens Medical Center, Athens, Greece; 4 Department of Vascular and Endovascular Surgery, Augusta Hospital, Duesseldorf, Germany

**Keywords:** lithotripsy, IVL, paclitaxel, DCB, PAD, femoropopliteal

## Abstract

**Introduction:**

The combination of intravascular lithotripsy (IVL) and drug-coated balloon (DCB) angioplasty for calcified peripheral lesions is associated with promising short-term results. However, data regarding the 12 months performance of this treatment option is missing. This study reports on the outcomes of IVL and DCB angioplasty for calcified femoropopliteal disease.

**Methods:**

Patients treated with IVL and DCB for calcified femoropopliteal lesions between February 2017 and September 2020 were included into this study. The primary outcome measure of this analysis was primary patency. Secondary patency, freedom from target lesion revascularization (TLR) and overall mortality were additionally analyzed.

**Results:**

Fifty-five (*n* = 55) patients and 71 lesions were analyzed. Most patients presented with long-term limb-threatening ischemia (*n* = 31, 56%), 47% (*n* = 26) were diabetics, and 66% (*n* = 36) had long-term kidney disease. The median lesion length was 77 mm (interquartile range: 45-136), and 20% (*n* = 14) of the lesions were chronic total occlusions (CTOs). Eccentric calcification was found in 23% of the vessels (*n* = 16), and circumferential calcium (peripheral arterial calcium scoring system [PACSS] Class 3 and 4) was present in 78% (*n* = 55) of the treated lesions.

The technical success after IVL amounted to 87% (*n* = 62) and the procedural success to 97% (*n* = 69). A flow-limiting dissection was observed in 2 cases (3%). Both the rates of target lesion perforation and distal embolization were 1% (*n* = 1). A bail-out scaffold was deployed in 5 lesions (7%). At 12 months the Kaplan-Meier estimate of primary patency was 81%, the freedom from TLR was 92% and the secondary patency 98%. The overall survival amounted to 89%, while the freedom from major amputation to 98%. The presence of eccentric disease, CTOs, or PACSS Class 4 did not increase the risk for loss of patency or TLR.

**Conclusions:**

In this challenging cohort of patients, the use of IVL and DCB for calcified femoropopliteal lesions was associated with promising 12 months outcomes and an excellent safety profile.

## Introduction

Endovascular treatment (ET) has been evolving rapidly the last years and is considered the first-line treatment strategy for patients with peripheral arterial disease (PAD).^1,2^ On the contrary, the presence of challenging lesions like chronic total occlusions (CTOs), calcified disease, and long lesions still limit the durability of minimally invasive procedures.^3–6^

Vascular calcification is associated with an increased risk for crossing failure and the stiffness of the arterial wall portends to dissections, recoil, and excessive injury after plain old balloon angioplasty (POBA).^3,7^ An additional challenge is the higher prevalence of calcified disease among high-risk subjects for cardiovascular morbidity and mortality, namely in patients with chronic kidney disease (CKD) or diabetes.^3,8^ Thus, both the effectiveness and the safety profile of a calcium-dedicated therapy are important parameters for the treatment of these frail patients.

Various endovascular modalities such as specialty balloons and atherectomy have been developed for the plaque modification of calcified vessels. Nonetheless, these treatment options primarily target the intimal calcium, and their use is associated with prolonged procedures and an elevated risk for complications.^9^ Intravascular lithotripsy (IVL), which uses pulsatile sonic waves to fracture intimal and medial calcium was recently introduced as a less-aggressive approach for the treatment of heavily calcified disease.^9–12^ Initial reports showed promising acute results with low complication rates and a clear preference of physicians toward a combined therapy with drug-coated balloons (DCBs) to inhibit restenosis.^9,10^ However, there is a paucity of data regarding the outcomes of IVL in combination with DCB for the treatment of peripheral vessels. The aim of this analysis is to assess the 12 months performance of IVL and DCB for severely calcified femoropopliteal disease.

## Methods

### Study Design

This study is a single-center, retrospective analysis of prospectively collected data, performed in line with the requirements of the local ethics committee and adhering to the declaration of Helsinki. All patients provided informed consent prior to the intervention.

Patients treated by IVL and DCB for calcified femoropopliteal disease between February 2017 and September 2020 were included into this study. Patients with in-stent-restenosis, aneurysm formation in the target lesion, isolated common/deep femoral artery disease or bypass anastomosis stenosis were excluded from this analysis. Patients treated with IVL and primary scaffolding or IVL as standalone therapy were additionally excluded. Patients with concomitant interventions due to aorto/iliac or infrapopliteal occlusive disease were not excluded from our analysis.

All patients underwent a thorough clinical examination at baseline. Patient demographics, comorbidities, imaging, and clinical data were prospectively collected and retrospectively analyzed. Follow-up examinations were scheduled at 6 and 12 months after the initial procedure and annually thereafter or in case of clinical worsening. The patency of the treated vessels was assessed by duplex ultrasound at each follow-up unless symptoms warranted angiography.

Dual antiplatelet therapy with aspirin (100 mg/d) and clopidogrel (75 mg/d) was routinely prescribed for 8 weeks, followed by aspirin or clopidogrel lifelong. Patients previously taking oral anticoagulants were maintained on the anticoagulant with an additional antiplatelet therapy for 8 weeks after the procedure. A triple therapy with dual antiplatelet medication and oral anticoagulation was not recommended. A statin therapy lifelong was suggested.

### Study Device and Procedural Protocol

The Shockwave Medical Peripheral IVL System (Shockwave Medical, Santa Clara, CA) consists of three parts: the generator, the connector cable, and the disposable IVL catheter. When activated, the generator transmits energy through the connector cable to the lithotripsy emitters of the IVL catheter. When the IVL catheter is inflated to low pressure (4 atm), a series of sonic pressure waves are produced, which then pass through the fluid-filled balloon and selectively disrupt the calcified plaque of the intima and/or media layers. Lithotripsy is administrated in 30-pulse cycles. After every cycle, the balloon is inflated to nominal pressure (6 atm) to maximize luminal gain. This cycle is repeated as needed and the catheter can be repositioned. The numbers of cycles applied was based on two parameters. The length of the lesion and the angiographic imaging after the first cycle. In “non-responding” lesions a second or third cycle was applied, or in some cases, the IVL catheter was activated in 6 atm. Sizing was performed based on preprocedural imaging (Duplex or CT scan) or in some cases on intravascular ultrasound measurements during the procedure. Vessel sizing is performed by measuring the distance from “media-to-media” in a relatively healthy vessel segment 1 cm proximal to the lesion. In case of a long lesion, a second measurement was performed 1 cm distally. A 1.1:1 ratio was used for the sizing of the IVL catheter and 1:1 for the DCB device.

The M5 peripheral IVL catheters house 5 lithotripsy emitters are 60 mm in length. The devices are available in multiple diameter sizes ranging from 3.5 to 7.0 mm in 0.5 mm increments and are compatible with a standard 0.014-inch guidewire. After crossing the lesion, a standard 0.014-inch guidewire was used to deliver the IVL catheter. A predilation with an undersized POBA catheter was performed in patients with CTOs or tight stenosis to enable the crossing of the IVL catheter. After the IVL treatment, adjunctive DCB therapy was applied to the entire lesion from “healthy-to-healthy” vessel with a DCB catheter of nominal diameter. The DCB selection was left to the discretion of the treating physician. In cases of a flow-limiting dissection or residual stenosis >50%, repeated prolonged (>3 minutes) POBA was applied. Provisional (bailout) stenting was used to treat major flow-limiting dissections or recoils (>30% restenosis after DCB angioplasty). A distal protection device was not routinely used, regardless the type of lesion

### Endpoints

The primary measure outcome of this study was primary patency, defined as freedom from significant restenosis or occlusion without any re-intervention. Secondary outcomes were secondary patency rate, freedom from clinically driven target lesion revascularization (TLR), amputation-free survival, and freedom from major amputation the overall survival.

### Definitions

Significant restenosis was indicated by a >2.0 peak systolic velocity ratio calculated as the peak systolic flow velocity in the lesion divided by the peak systolic velocity 1 cm proximal to the lesion. Secondary patency was defined as restored flow in the treated segment after occlusion or restenosis. Amputation-free survival was defined as the time until a major amputation of the index limb and/or death of any cause, whichever occurred first. A major amputation was defined as any above-ankle amputation. Procedural technical success was defined as residual stenosis <30% in the absence of arterial perforation of the treated segment. Technical success after IVL was defined as residual stenosis <50% in the absence of arterial perforation and flow-limiting dissection. The calcification burden was graded on the basis of the arterial wall calcium deposits observed during fluoroscopy based on the Peripheral Arterial Calcium Scoring Scale (PACSS). Grade 0 represents the lack of visible calcium at the target lesion, grade 1 refers to unilateral calcification shorter than 5 cm, grade 2 refers to unilateral wall calcification longer than 5 cm, grade 3 shows the presence of bilateral wall calcification shorter than 5 cm, and finally, grade 4 is defined as bilateral wall calcification with calcium extension longer than 5 cm.^13^ Six grades of dissection (A-F) were identified. Type A was defined as dissection with minor radiolucent areas, type B as linear dissection, type C as dissection with contrast agent outside the lumen, type D as spiral dissection, type E as persistent filling defects, and F as vessel occlusion without distal antegrade flow. Severe vessel dissection patterns were defined as type C or higher.

### Statistical Analysis

For the statistical analysis and graphics, the MedCalc Statistical Software (version 12.4.0.0; MedCalc Software, Ostend, Belgium) was used. Continuous variables are presented as means ± standard deviation or median (interquartile range), while categorical data are given as the counts. Continuous numeric variables were compared by Student *t* test for paired samples or Wilcoxon test according to their distribution (D-Agostino-Pearson test). Cumulative primary and secondary patency, as well as freedom from TLR and amputation-free survival were estimated using the Kaplan-Meier method. A univariate analysis was performed for each outcome (patients with patent vessels vs patients with patency loss and patients with TLR versus patients without TLR) to identify statistically significant differences between the groups. These variables were included in Cox regression analyses to determine risk factors for patency loss and TLR, respectively. The threshold of statistical significance was *p* ≤ .05.

## Results

### Baseline Characteristics

Fifty-five patients (*n* = 55) with 71 calcified femoropopliteal lesions were included into this analysis. Most patients (*n* = 31, 56%) presented with long-term limb-threatening ischemia (Rutherford class 4-6), 47% (*n* = 26) were diabetics, and 66% (*n* = 36) had CKD. The mean preoperative ankle brachial index (ABI) amounted to 0.64 ± 0.41. Table 1 provides an overview of the baseline characteristics of the study population.

**Table 1.** Patients Characteristics.

Characteristics	Results
Total number of patients	55
Males	27 (49%)
Mean age (±SD), in years	75 ± 8
Arterial hypertension	51 (93%)
Dyslipidemia	42 (76%)
Diabetes mellitus	26 (47%)
Congestive heart disease	30 (55%)
Chronic kidney disease	36 (66%)
End-stage renal disease	3 (6%)
Cerebrovascular disease	10 (18%)
Smoking (former)	4 (7%)
Smoking (current)	8 (15%)
Rutherford classes	
Class 3	24 (44%)
Class 4	13 (24%)
Class 5	14 (26%)
Class 6	4 (7%)
Long-term limb-threatening ischemia	31 (56%)
Mean ABI (±SD)	0.64 ± 0.41
Previous intervention	29 (53%)

Abbreviation: ABI, ankle brachial index.

The median lesion length was 77 mm (interquartile range [IQR]:45-136) and 20% (*n* = 14) of the lesions were CTOs. Regarding the severity of calcification, 2 lesions were classified as PACSS 1 (*n* = 3%), 13% of the lesions were PACSS class 2 (*n* = 9), 47% PACSS class 3 (*n* = 33), and 31% PACSS 4 (*n* = 22). Eccentric calcification was found in 23% of the vessels (*n* = 16). Popliteal artery involvement was observed in 26 (47%) individuals. In 15 patients, a single tibial vessel was patent, in 21 patients, 2 vessels were patent, while in 19 patients, all three tibial arteries were patent.

### Early Outcomes

The post-IVL technical success and the procedural technical success rates amounted to 87% (*n* = 62) and 97% (*n* = 69), respectively. The median diameter post-IVL was 5.1 (4.6-5.8) mm. The rates of both target lesions perforation and peripheral embolization were 1% (*n* = 1). A bail-out scaffold was deployed in 5 lesions (7%). The rate of flow-limiting was 3% (*n* = 2). The median postoperative ABI at discharge was 1 (IQR: 0.93-1.00). A high-dose DCB (IN.PACT Admiral, Medtronic, Dublin, Ireland) was used in 42 lesions (59%), while a low-dose DCB catheter (Stellarex, Phillips/Spectranetics Corp., Colorado Springs, CO and Ranger, Boston Scientific, Marlborough, Massachusetts) was preferred in 29 lesions (31%). Table 2 summarizes the procedural characteristics and the in-hospital outcomes.

**Table 2.** Procedural Characteristics.

Characteristics	Results
Inflow intervention	9 (13%)
Concomitant femoropopliteal intervention	17 (25%)
Concomitant crural/pedal intervention	12 (17%)
IVL oversized	48 (68%)
DCB high dose	42 (59%)
Procedural technical success	69 (97%)
Technical success after IVL	62 (87%)
Perforation	1 (1%)
Peripheral embolization	1 (1%)
Flow-limiting dissection	2 (3%)
Non-flow-limiting dissection	3 (4%)
Bail-out stenting	5 (7%)
Reintervention before discharge	0
Median ABI at discharge (IQR)	1.00 (0.93-1.00)
Median length of hospital stay (IQR), in days	2 (2-4)

Abbreviations: ABI, ankle brachial index; DCB, drug-coated balloon; IVL, intravascular lithotripsy; IQR, interquartile range.

### Outcomes in Follow-Up

**Figure 1.**
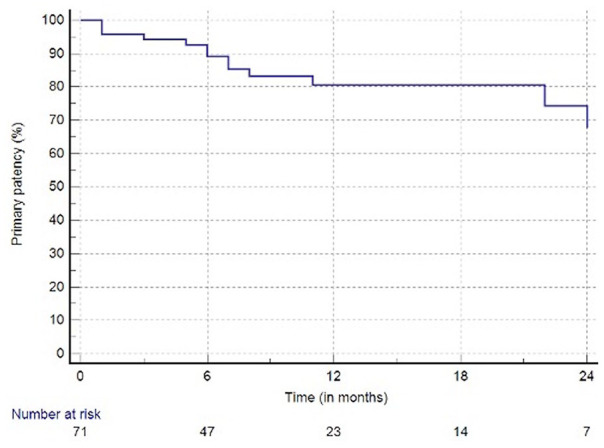
Primary patency of intravascular lithotripsy and drug-coated balloon angioplasty at 12 months 81% (SE > 10% at 24 months).

The mean follow-up time was 12 months (SD ±9). The primary patency at 12 months amounted to 81% (95% confidence interval[CI]: 70%-90%) (Figure 1). The freedom from TLR at 12 months was 92% (95% CI: 86%-99%) and the secondary patency rate 98% (95% CI: 95%-100%). The amputation-free survival rate was 89% (95% CI: 81%-97%), the freedom from major amputation was 98% (95% CI: 95%-100%) and the overall survival rate amounted to 89% at 12 months. Regarding the clinical status of the study cohort at the last follow-up, most patients (*n* = 44 patients, 88%) were either asymptomatic or complained about claudication (Class 1-3), 4 patients (7%) had ischemic rest pain (class 4), and 7 patients (13%) had persistent tissue loss (Class 5). For the Cox-regression analysis, univariate analyses between patients with and without patency loss and with and without TLR was performed. The presence of eccentric disease, CTOs, or PACSS 4 lesions did not increase the risk for patency loss or TLR, while the use of oversized IVL catheters or high-dose DCBs did not influence the outcomes of the combination therapy. Finally, distal popliteal artery disease increased the risk for patency loss. (Table 3)

**Table 3.** Cox Regression Analysis for Patency Loss and TLR.

Target lesion revascularization			
Covariate	TLR	No TLR	*p*
SFA	35 (92%)	3 (8%)	.858
SFA proximal	17 (26%)	0	.154
SFA middle	21 (96%)	1 (4%)	.431
SFA distal	27 (93%)	2 (7%)	.670
Popliteal proximal (P1)	18 (86%)	3 (14%)	.267
Popliteal middle (P2)	20 (95%)	1 (5%)	.472
Popliteal distal (P3)	4 (6%)	1 (17%)	.339
PACSS 3	29 (88%)	4 (12%)	.304
PACSS 4	22 (100%)	0	.089
CTO	14 (100%)	0	.208
Lesion length	64	54	.601
Eccentric	15 (94%)	1 (6%)	.721
IVL oversizing	44 (92%)	4 (8%)	.959
DCB high dose	39 (93%)	3 (50%)	.636
Patency loss			
Covariate	Patent	Patency loss	*p*
SFA	32 (84%)	6 (16%)	.375
SFA proximal	16 (94%)	1 (7%)	.103
SFA middle	18 (82%)	4 (18%)	.829
SFA distal	25 (86%)	4 (14%)	.301
Poplitea proximal (P1)	14 (67%)	7 (33%)	.070
Poplitea middle (P2)	16 (76%)	5 (24%)	.577
Poplitea distal (P3)	2 (40%)	3 (60%)	**.020**
PACSS 3	25 (76%)	8 (24%)	.375
PACSS 4	20 (91%)	2 (9%)	.134
CTO	13 (93%)	1 (7%)	.190
Lesion length	66	58	.191
Eccentric	13 (81%)	3 (19%)	.913
IVL oversizing	38 (79%)	10 (21%)	.735
High-dose DCB	35 (83%)	7 (17%)	.440
Cox regression	HR	95%CI	p
P3	6.12	1.61-23.19	.008

Abbreviations: CTO, chronic total occlusion; DCB, drug-coated balloon; IVL, intravascular lithotripsy; PACSS, peripheral arterial calcium scoring system; SFA, superficial femoral artery.

## Discussion

Arterial wall calcification impedes the ET of PAD by multiple mechanisms including crossing failure, insufficient luminal gain, and loss of patency. The aim of a calcium-dedicated treatment strategy is to effectively modify the plaque without increasing the risk for periprocedural complications in these frail patients. In this study, the combination of IVL and DCB angioplasty was associated with high technical- and procedural-success rates, low risk for peri-interventional complications and bail out stenting and promising outcomes at 12 months.

The use of IVL as standalone therapy for the treatment of peripheral lesions was evaluated in the framework of two prospective trials. The DISRUPT BTK was a single-arm, multicenter, feasibility, and safety study, which assessed the acute results of IVL in infrapopliteal lesions. The study enrolled 20 patients and no major adverse event, TLR or amputation was reported at 30 days. Technical success (≤50% residual stenosis) was achieved in all procedures, a single non-flow-limiting dissection was observed, and none of the subjects experienced thrombosis or distal embolization.^11^ The DISRUPT PAD II trial was a nonrandomized, multicenter study which evaluated the performance of IVL in calcified femoropopliteal disease. This trial enrolled a total of 60 subjects (all claudicants), the average lesion length was 76.9 mm and 16.7% were CTOs. Similar to the BKT cohort, a very low periprocedural complication rate was observed. However, the use of IVL was associated with an increased risk for treatment failure and repeated revascularization as the rates of primary patency and TLR at 12 months were 54.5% and 20.7%, respectively. An important finding of this study was that the optimal IVL technique (1.1:1 oversizing and avoidance of therapeutic miss) improved the primary patency to 62.9% and reduced the TLR rate to 8.6%.^12^ Given the high rates of patency loss and TLR after IVL as standalone therapy, the adjunctive use of an antirestenotic treatment might be beneficial.

Paclitaxel is still the main antiproliferative agent used for peripheral interventions. However, despite the promising results of DCBs in short, fibrotic lesions, the ability of paclitaxel to inhibit restenosis in severely calcified disease remains debatable. Fanelli et al reported an increased rate of patency loss in case of circumferential calcium, while in a retrospective study of 91 patients, the presence of severe calcification was associated with an increased late lumen loss.^14,15^ In this analysis, the use of DCB after IVL led to improved results compared to the reported data of IVL as standalone modality. The ongoing DISRUTP PAD III trial (NCT02923193) will further evaluate the performance of the combination therapy. This study randomized 306 patients to POBA or IVL prior to DCB angioplasty. The 30 days outcomes showed a higher procedural success in the IVL group and a significant reduction in the frequency and severity of dissections.^16^ Nonetheless, the exclusion of patients with advanced ischemia (Rutherford class 5 and 6) and impaired renal function as well as the use of a control group (POBA and DCB) which is associated with an increased risk for clinical failure might limit the clinical relevance of this study. Of note, most patients in our analysis presented with CLTI and/or CKD.

The use of interwoven stents, stent grafts, and different “leave-nothing-behind” approaches has been recently described for the treatment of calcified femoropopliteal disease.^3^ In a small cohort of 34 patients,the use of the Supera platform (Abbott Vascular, Santa Clara, CA, USA) in long (mean lesion length 27.9 cm), moderate- and severely calcified lesions led to a primary patency of 94.1%.^17^ Moreover, in a retrospective analysis of 67 patients with long, heavily calcified disease (mean lesion length 26.9 cm, 62% PACSS 4), the “pave-and-crack” technique with the Supera scaffold and the Viabahn stent-graft (W.L. Gore & Associates, Flagstaff, AZ, USA) was associated with a primary patency of 79%.^18^ The increased radial force of the interwoven stents can address the challenges raised from calcium. However, the vessel preparation prior to Supera deployment is an important factor for the durability of the reconstruction, while the aggressive predilatation often required might increase the risk for complications and pseudoaneurysm formation.^17,19^ In this context and given the promising initial experience of IVL in combination with scaffolds for coronary disease, a potential benefit from IVL prior to stent deployment should be evaluated.^20^

Furthermore, studies evaluating atherectomy or specialty balloons prior to DCB angioplasty for femoropopliteal lesions showed controversial results. In the DEFINITIVE AR trial, a trend to improved outcomes in calcified lesions was observed following directional atherectomy and anti-restenotic therapy compared to DCB alone.^21^ The REALITY (Directional atherectomy plus drug-coated balloon to treat long, calcified femoropopliteal artery lesions) trial prospectively enrolled 102 subjects with long calcified lesions (mean lesion length 22.6±8.6cm, 86% moderate to severe bilateral calcification). At 12 months the primary patency rate after directional atherectomy and DCB was 77% and the freedom from TLR was 93%.^22^ On the other hand, in two retrospective registries, the use of orbital atherectomy or scoring balloons did not significantly improve the outcomes of DCB angioplasty.^23,24^ Although atherectomy seems to be an effective treatment strategy, debulking in long calcified lesions increases the procedural time, necessitates repeated angiograms and higher volumes of contrast agent. Thus, the applicability of these techniques in older patients or subjects with CKD might be limited. Although no comparison trial is available, the reduced risk for embolic complications following the use of IVL might offer an advantage over atherectomy in cases with poor run off.

**Figure 2.**
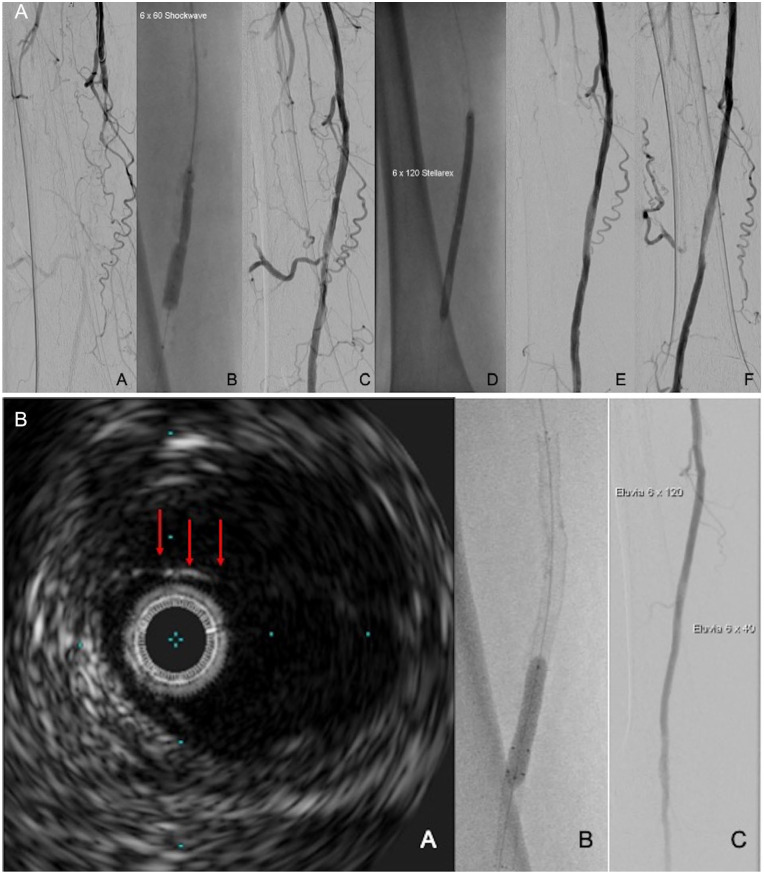
Intravascular ultrasound guided intravascular lithotripsy (IVL) and drug-coated balloon (DCB) angioplasty: (A) A: calcified femoropopliteal occlusion, B: IVL angioplasty, C: angiogram after IVL, D: DCB angioplasty, E, F: angiogram post-IVL and DCB in two planes with no evidence of a significant dissection and (B) A: intravascular ultrasound revealing a dissection (arrows) of the treated segment, B, C: Eluvia drug eluting stent deployment (Boston Sci.) to treat the dissection.

Similar to our results, a constant finding among all studies reporting on peripheral IVL is the high technical success—the decreased needed for scaffolds and the low periprocedural complications—rates.^9–12^ Even though these parameters are important in the treatment of this challenging cohort, the long-term performance of IVL in combination with DCB or scaffolds has to be assessed in large-scale real-world studies. In addition, a slight oversizing (0.5 cm) of the IVL catheter is suggested. This might be, however, problematic in long femoropopliteal lesions with different vessel diameters between the proximal and the distal part of the artery. An additional limitation for the treatment of very long lesions is that the M5 catheter can deliver only 300 pulses. In these cases, the use of a second device should be considered; however, at the cost of increased procedural expenses. Finally, and similar to the coronaries, an intravascular imaging guided treatment might improve the outcomes of peripheral IVL.^25^ Intravascular ultrasound is shown to be superior to angiography for the estimation of the vessel diameter and consequently might be helpful for the appropriate IVL catheter.^26^ It can also detect early recoils or dissections, which are not always visible with conventional imaging (Figure 2).

### Limitations

Although, this is the first analysis reporting on the outcomes of peripheral IVL and DCB angioplasty for calcified femoropopliteal disease, this study carries the well-known limitations of registries. The retrospective nature of this study, the lack of a control group, and the absence of core laboratory adjudication are further limitations of this study. In addition, during the study period, and similar to the DISRUPT PAD III trial, the IVL generator was modified, enabling the delivery of 300 pulses instead of 180 pulses with the initial device. Furthermore, the DCB selection was at the discretion of the treating physician. The use of high-dose devices did not influence the performance of the combination therapy, it is, however, possible that the use of different DCB catheters might alter the observed results. No toe pressure measurements were performed in case of false-elevated ABI. Finally, although most patients were asymptomatic at last follow-up, data regarding time-to-wound healing were not collected and accordingly cannot be analyzed.

## Conclusions

In this study, the use of IVL in combination with DCB showed promising acute outcomes and an excellent safety profile for the treatment of severely calcified disease. Despite the inclusion of patients with increased comorbidity, the combination therapy was associated with acceptable results and low rates of reinterventions at 12 months. The optimal use of IVL and the combination of IVL with scaffolds needs to be evaluated in the framework of prospective real-world studies.

## References

[1] Conte MS Bradbury AW Kolh P , et al. Global vascular guidelines on the management of chronic limb-threatening ischemia. J Vasc Surg. 2019;69(6S):3S–125S.e40. 10.1016/j.jvs.2019.02.016 PMC8365864 31159978

[2] Stavroulakis K Borowski M Torsello G , et al. One-year results of first-line treatment strategies in patients with critical limb ischemia (CRITISCH registry). J Endovasc Ther. 2018;25(3):320–329. 29968501 10.1177/1526602818771383

[3] Stavroulakis K Argyriou A Watts M , et al. How to deal with calcium in the superficial femoral artery. J Cardiovasc Surg (Torino). 2019;60(5):572–581. 10.23736/S0021-9509.19.11038-5 31241269

[bibr4-15266028221075563] Torsello G Stavroulakis K Brodmann M , et al. Three-year sustained clinical efficacy of drug-coated balloon angioplasty in a real-world femoropopliteal cohort. J Endovasc Ther. 2020;27(5):693–705. 32583749 10.1177/1526602820931477 PMC7545651

[bibr5-15266028221075563] Patel MR Conte MS Cutlip DE , et al. Evaluation and treatment of patients with lower extremity peripheral artery disease: consensus definitions from Peripheral Academic Research Consortium (PARC). J Am Coll Cardiol. 2015;65(9):931–941. 25744011 10.1016/j.jacc.2014.12.036 PMC4874808

[6] Okuno S Iida O Shiraki T , et al. Impact of calcification on clinical outcomes after endovascular therapy for superficial femoral artery disease: assessment using the peripheral artery calcification scoring system. J Endovasc Ther. 2016;23(5):731–737. 27369975 10.1177/1526602816656612

[7] Saab F Jaff MR Diaz-Sandoval LJ , et al. Chronic total occlusion crossing approach based on plaque cap morphology: the CTOP classification. J Endovasc Ther. 2018;25(3):284–291. 29484959 10.1177/1526602818759333

[8] Karimi Galougahi K Patel S Shlofmitz RA , et al. Calcific plaque modification by acoustic shock waves: intravascular lithotripsy in coronary interventions. Circ Cardiovasc Interv. 2021;14(1):e009354. 32907343 10.1161/CIRCINTERVENTIONS.120.009354

[9] Madhavan MV Shahim B Mena-Hurtado C , et al. Efficacy and safety of intravascular lithotripsy for the treatment of peripheral arterial disease: an individual patient-level pooled data analysis. Catheter Cardiovasc Interv. 2020;95(5):959–968. 31957955 10.1002/ccd.28729 PMC7187419

[bibr10-15266028221075563] Adams G Shammas N Mangalmurti S , et al. Intravascular lithotripsy for treatment of calcified lower extremity arterial stenosis: initial analysis of the disrupt PAD III study. J Endovasc Ther. 2020;27(3):473–480. 32242768 10.1177/1526602820914598 PMC7288854

[bibr11-15266028221075563] Brodmann M Holden A Zeller T. Safety and feasibility of intravascular lithotripsy for treatment of below-the-knee arterial stenoses. J Endovasc Ther. 2018;25(4):499–503. 29911480 10.1177/1526602818783989 PMC6041733

[12] Brodmann M Werner M Holden A , et al. Primary outcomes and mechanism of action of intravascular lithotripsy in calcified, femoropopliteal lesions: results of Disrupt PAD II. Catheter Cardiovasc Interv. 2019;93(2):335–342. 30474206 10.1002/ccd.27943

[13] Rocha-Singh KJ Zeller T Jaff MR. Peripheral arterial calcification: prevalence, mechanism, detection, and clinical implications. Catheter Cardiovasc Interv. 2014;83:e212–e220. 24402839 10.1002/ccd.25387 PMC4262070

[14] Fanelli F Cannavale A Gazzetti M , et al. Calcium burden assessment and impact on drug-eluting balloons in peripheral arterial disease. Cardiovasc Intervent Radiol. 2014;37(4):898–907. 24806955 10.1007/s00270-014-0904-3

[15] Tepe G Beschorner U Ruether C , et al. Drug-eluting balloon therapy for femoropopliteal occlusive disease: predictors of outcome with a special emphasis on calcium. J Endovasc Ther. 2015;22(5):727–733. 26250747 10.1177/1526602815600156

[16] Gray WA. IVL for peripheral artery calcium: the DISRUPT PAD III randomized controlled trial 30-day outcomes. Presented at: VIVA 2020. November 7, 2020; Las Vegas.

[17] Palena LM Diaz-Sandoval LJ Sultato E , et al. Feasibility and 1-Year outcomes of subintimal revascularization with supera® stenting of long femoropopliteal occlusions in critical limb ischemia: the “Supersub” Study. Catheter Cardiovasc Interv. 2017;89(5):910–920. 27862880 10.1002/ccd.26863

[18] Dias-Neto M Matschuck M Bausback Y , et al. Endovascular treatment of severely calcified femoropopliteal lesions using the “pave-and-crack” technique: technical description and 12-month results. J Endovasc Ther. 2018;25(3):334–342. 29557221 10.1177/1526602818763352

[19] Garcia LA Rosenfield KR Metzger CD , et al. SUPERB final 3-year outcomes using interwoven nitinol biomimetic supera stent. Catheter Cardiovasc Interv. 2017;89(7):1259–1267. 28471091 10.1002/ccd.27058

[20] Kereiakes DJ Hill JM Ben-Yehuda O , et al. Evaluation of safety and efficacy of coronary intravascular lithotripsy for treatment of severely calcified coronary stenoses: design and rationale for the Disrupt CAD III trial. Am Heart J. 2020;225:10–18. 32470635 10.1016/j.ahj.2020.04.005

[21] Zeller T Langhoff R Rocha-Singh KJ , et al. Directional atherectomy followed by a paclitaxel-coated balloon to inhibit restenosis and maintain vessel patency: twelve-month results of the DEFINITIVE AR study. Circ Cardiovasc Interv. 2017;10(9):e004848. 28916599 10.1161/CIRCINTERVENTIONS.116.004848 PMC5610565

[22] Rocha-Singh KJ. REALITY Study: directional atherectomy vessel preparation prior to DCB angioplasty. Presented at: VIVA 2020. November 7, 2020; Las Vegas.

[23] Foley TR Cotter RP Kokkinidis DG , et al. Mid-term outcomes of orbital atherectomy combined with drug-coated balloon angioplasty for treatment of femoropopliteal disease. Catheter Cardiovasc Interv. 2017;89(6):1078–1085. 28295971 10.1002/ccd.26984

[24] Lugenbiel I Grebner M Zhou Q , et al. Treatment of femoropopliteal lesions with the AngioSculpt scoring balloon—results from the Heidelberg PANTHER registry. Vasa. 2018;47(1):49–55. 29116910 10.1024/0301-1526/a000671

[25] Mattesini A Nardi G Martellini A , et al. Intravascular imaging to guide lithotripsy in concentric and eccentric calcific coronary lesions. Cardiovasc Revasc Med. 2020;21(9):1099–1105. 32471713 10.1016/j.carrev.2020.04.016

[26] Pliagas G Saab F Stavroulakis K , et al. Intravascular ultrasound imaging versus digital subtraction angiography in patients with peripheral vascular disease. J Invasive Cardiol. 2020;32(3):99–103. 32123141 10.25270/jic/19.00418

